# Piezo1-induced durotaxis of pancreatic stellate cells depends on TRPC1 and TRPV4 channels

**DOI:** 10.1242/jcs.263846

**Published:** 2025-04-25

**Authors:** Ilka Budde, André Schlichting, David Ing, Sandra Schimmelpfennig, Anna Kuntze, Benedikt Fels, Joelle M.-J. Romac, Sandip M. Swain, Rodger A. Liddle, Angela Stevens, Albrecht Schwab, Zoltán Pethő

**Affiliations:** ^1^ Institute of Physiology II, University of Münster, Robert-Koch Str. 27B, 48149 Münster, Germany; ^2^ Institute for Analysis and Numerics, University of Münster, Einsteinstr. 62, 48149 Münster, Germany; ^3^ Institute of Applied Analysis, University of Ulm, Helmholtzstraße 18, 89081 Ulm, Germany; ^4^ Gerhard-Domagk-Institute of Pathology, University of Münster, 48149 Münster, Germany; ^5^ Institute of Physiology, University of Lübeck, 23562 Lübeck, Germany; ^6^ Department of Medicine, Duke University, Durham, NC 27708, USA

**Keywords:** Mechanosensation, Mechanosignaling, Pancreatic cancer, Taxis

## Abstract

Pancreatic stellate cells (PSCs) are primarily responsible for producing the stiff tumor tissue in pancreatic ductal adenocarcinoma (PDAC). Thereby, PSCs generate a stiffness gradient between the healthy pancreas and the tumor. This gradient induces durotaxis, a form of directional cell migration driven by differential stiffness. However, the molecular sensors behind durotaxis are still unclear. To investigate the role of mechanosensitive ion channels in PSC durotaxis, we established a two-dimensional stiffness gradient mimicking PDAC. Using pharmacological and genetic methods, we investigated the contribution of the ion channels Piezo1, TRPC1 and TRPV4 in PSC durotaxis. We found that PSC migration towards a stiffer substrate is diminished by altering Piezo1 activity. Moreover, disrupting TRPC1 along with TRPV4 abolishes PSC durotaxis even when Piezo1 is functional. Our results demonstrate that optimal PSC durotaxis requires an intermediary level of ion channel activity, which we simulated via a numerically discretized mathematical model. These findings suggest that mechanosensitive Piezo1 channels detect the differential stiffness microenvironment. The resulting intracellular signals are amplified by TRPV4 and TRPC1 channels to guide efficient PSC durotaxis.

## Teaser

**Summary:** Cells move to areas of higher stiffness via a process called durotaxis. We show that pancreatic stellate cell durotaxis, which might be relevant in pancreatic cancer, is orchestrated by Piezo1, TRPV4 and TRPC1 channels.

## INTRODUCTION

Increased tissue stiffness is characteristic of human solid tumors. Higher stiffness is usually associated with more aggressive tumor growth and, thus, a poorer prognosis (Piersma et al., 2020). Among solid tumors, pancreatic ductal adenocarcinoma (PDAC) has an exceptionally stiff (desmoplastic) stroma. A consequence of desmoplasia is an associated increase in tissue stiffness, measurable by atomic force microscopy (AFM) or magnetic resonance elastography. Whereas healthy pancreatic tissue has an average stiffness of 1–2 kPa, PDAC stiffness ranges between 5 kPa and 10 kPa (Rubiano et al., 2018; Shi et al., 2018). These differences create a stiffness gradient between the soft, healthy pancreas and the stiff, desmoplastic tumor.

The stromal components of PDAC can account for up to 80% of the tumor mass (Apte et al., 2015). The tumor stroma enhances PDAC malignancy in various ways: (1) pancreatic stellate cells (PSCs) produce excess extracellular matrix (ECM) (Pothula et al., 2020); (2) the resulting increase in stiffness further activates and/or maintains the activation of PSCs and (3) compresses blood vessels, leading to (4) reduced blood flow, thus abolishing the efficacy of chemotherapy (Apte et al., 2015); (5) carcinoma cells activate PSCs, and reciprocally, PSCs promote cancer cell proliferation, migration and survival (Apte et al., 2015); and (6) PSCs can self-activate in an autocrine manner (Pothula et al., 2020; Pethő et al., 2019; Nielsen et al., 2017). Therefore, PSCs and other cancer-associated fibroblasts (CAFs) create and maintain the tumor-promoting microenvironment. Notably, the characteristic mechanical properties of the PDAC microenvironment maintain a positive mechanical feedback loop that further promotes fibrosis (Lachowski et al., 2017).

PSCs sense the stiffness difference between PDAC and healthy pancreatic tissue by migrating toward the stiffer areas (Lachowski et al., 2017). Directional migration along stiffness gradients of the ECM is referred to as durotaxis (Espina et al., 2022). Durotaxis is also found in other human cell types, including fibroblasts, carcinoma cells and smooth muscle cells (Lo et al., 2000; DuChez et al., 2019; Evans et al., 2018). Although various elements of the migration machinery are well documented, the primary mechanosensory transduction process that enables (pancreatic stellate) cells to respond to stiffness gradients still needs to be clarified (Lachowski et al., 2018, 2017; DuChez et al., 2019; Janmey et al., 2020; Espina et al., 2022).

Since cells can respond to mechanical stimuli in their environment, they must possess appropriate sensory mechanisms. Several potential mechanosensory and mechanotransduction pathways exist involving integrins, focal adhesion complexes, G-protein-coupled receptors and mechanosensitive ion channels (Espina et al., 2022). Many ion channels involved in mechanosignaling are Ca^2+^ permeable and are close to focal adhesions (Kobayashi and Sokabe, 2010; Yao et al., 2022; Ellefsen et al., 2019). They can detect mechanical stresses and/or translate them into a Ca^2+^ influx. Thus, Ca^2+^ is a central second messenger that can initiate global cellular responses to a local mechanical event (Nourse and Pathak, 2017). However, the link between mechanosensation and the initiation of the corresponding cellular response still needs to be understood.

The most prominent mechanosensitive ion channel is Piezo1, whereas TRPV4 and TRPC1 channels are involved in downstream mechanosignaling events (Coste et al., 2010; Goswami et al., 2010; Swain et al., 2022). Piezo1 is expressed in PSCs and is involved in cell migration in a pH-dependent manner (Fels et al., 2016; Kuntze et al., 2020). Moreover, Piezo1 and TRPV4 play a role in pressure-induced pancreatitis, which might act as a precursor lesion for PDAC (Swain et al., 2020). Additionally, other ion channels expressed in PSCs might also be involved in an indirect manner, such as TRPC1 and K_Ca_3.1 (encoded by *KCNN4*) (Storck et al., 2017; Fabian et al., 2012). TRPC1 confers the pressure-induced activation of PSCs (Fels et al., 2016; Radoslavova et al., 2022). The expression pattern of the ion channels also shows a reciprocal influence: knockout (KO) of the TRPC1 channel in PSCs leads to overexpression of the TRPV4 channel (Fels et al., 2016).

Whether mechanosensitive ion channels in PSCs play a role in stiffness gradient perception has not yet been explored. Ion channels are key integration sites for polymodal extracellular stimuli (Pethő et al., 2019). Thus, we hypothesized that they act as a ‘missing link’ between ECM stiffness perception and durotaxis. This work aims to show the extent to which ion channels mediate PSC durotaxis.

## RESULTS

### PSC phenotype is altered in a stiffness-dependent manner

**Fig. 1.**
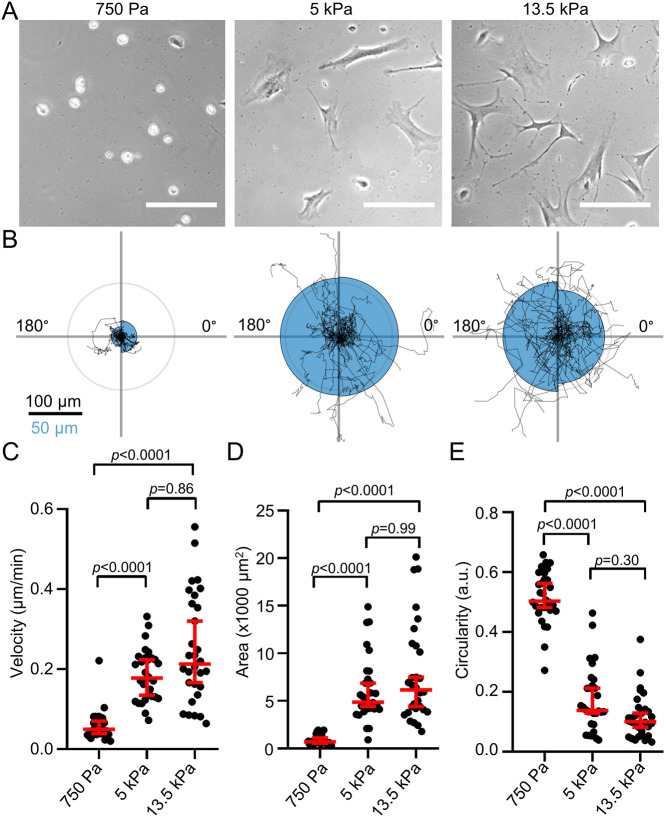
**Substrate stiffness affects PSC migration and morphology.** (A) Representative phase-contrast microscopy images of PSCs seeded on substrates with different stiffnesses, as indicated. Scale bars: 100 µm (B) Durotaxis polar plots depict individual PSC trajectories over 24 h (black lines) on substrates with stiffnesses as indicated for the images above in A. The radii of the blue half circles on the right-hand and left-hand sides of each plot are proportional to the mean cellular displacement towards 0° and 180°, respectively. Radial lines indicate 0°, 90°, 180° and 270°. Scale bar: 100 µm for the migration trajectories and 50 µm for the half circles. The radius of the concentric gray circle is a visual aid for the scale bar. (C–E) Scatter plots depict PSC velocity (C), area (D) and circularity (E) on substrates with indicated stiffness levels (*n*=30 cells from *N*=3 experiments). Data and statistical comparisons in C–E: median±95% confidence interval; Kruskal–Wallis statistical test with Dunn's post-hoc test. a.u., arbitrary units.

First, we tested whether the phenotype of PSCs differs when they are seeded on soft and stiff matrices reflecting the properties of the healthy pancreas and desmoplastic PDAC, respectively. For this, we created polyacrylamide hydrogels (Table S1) coated with a thin layer of ECM. These hydrogels accurately mimic the substrate stiffness levels of the healthy pancreas (750 Pa) and the increased stiffness seen in PDAC (5 kPa), as well as an even greater stiffness (13.5 kPa) (Fig. S1A) (Rubiano et al., 2018; Shi et al., 2018). We seeded murine PSCs onto the hydrogels and observed their two-dimensional phenotype (Fig. 1A) and migratory behavior (Fig. 1B). The durotaxis plots show that PSCs have no preferential directionality without a stiffness gradient (Fig. S1B). PSCs migrate substantially more slowly on a soft 750 Pa substrate than cells on stiffer substrates (Fig. 1C). Moreover, PSCs spread less on softer substrates. PSCs seeded onto a 750 Pa hydrogel have ∼10 times less area (Fig. 1D) and a fivefold higher circularity (Fig. 1E) compared to PSCs on 5 kPa and 13.5 kPa substrates. Cells seeded onto 5 kPa and 13.5 kPa substrates behave similarly, suggesting that PSC spreading approaches a maximum and cannot be further increased by higher stiffness values.

### PSCs undergo durotaxis under pathophysiologically relevant stiffness conditions

**Fig. 2.**
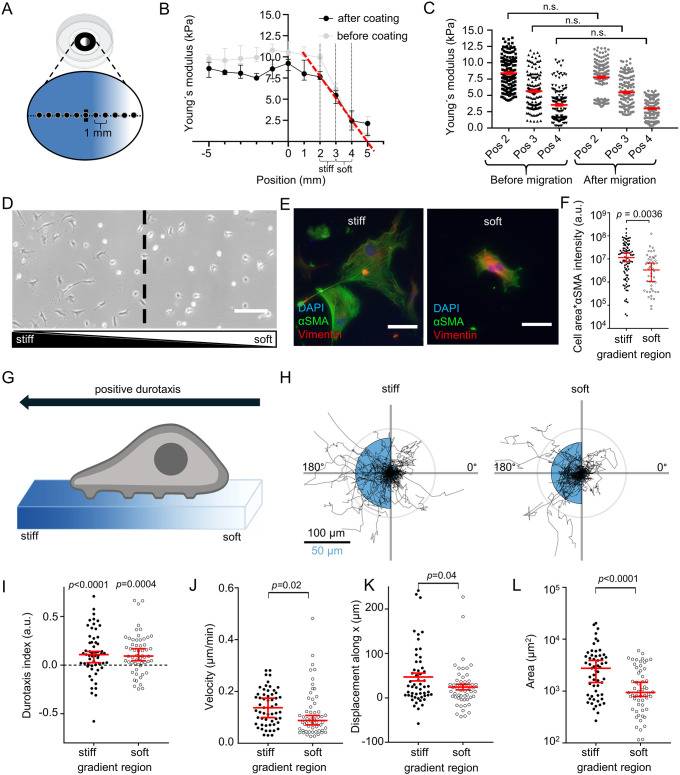
**PSCs undergo durotaxis on linear stiffness gradient hydrogels.** (A) Experimental setup of gradient gel construction. Glass-bottom dishes were coated with a UV polymerized hydrogel using a gradient photomask (grayscale form inside the dish). The resulting stiffness gradient of the gel was measured with AFM from stiff (blue) to soft (white), starting from the middle of the gel (horizontal line) in 1 mm steps (black dots on line). Created in BioRender by Pethö, Z., 2025. https://BioRender.com/eaiqsui. This figure was sublicensed under CC-BY 4.0 terms. (B) Gradient hydrogel stiffness before (gray) and after (black) ECM coating. *n*=40 points measured across *N*=4 gels. The stiffest area of the gradient hydrogel (position 2 mm, ‘stiff’) has a median stiffness of 7.6 kPa (95% confidence interval: 7.3–8.3 kPa), whereas the soft area of the gel (position 4 mm, ‘soft’), has a median stiffness of 2.4 kPa (95% confidence interval: 1.3–3.6 kPa). Note the linear stiffness decay in the gradient region after coating (red dashed line, slope=−2.47 kPa/mm). (C) Scatter plots depict gel stiffness measured before (black) and after (gray) cell migration assays at the indicated position (Pos). *n*=33 points measured on each of *N*=3 gels. (D) Representative phase-contrast image of PSCs on an ECM-coated gradient hydrogel. The dashed line indicates the center of the gradient separating ‘stiff’ and ‘soft’ gradient regions. Scale bar: 250 µm. Image representative of *N*=4 experiments. (E) Representative immunofluorescence images of the myofibroblastic PSC marker αSMA (green), vimentin (red) and DAPI (blue) in PSCs seeded on stiff (left) and soft (right) regions. Images are representative of *N*=4 experiments. Scale bars: 150 µm. (F) Scatter plot of total PSC αSMA fluorescence assessed by multiplying cell area with αSMA fluorescence intensity. *n*=51 cells on a soft substrate and *n*=99 cells on a stiff substrate were measured across *N*=4 experiments. (G) Schematic illustration of ‘positive’ durotaxis: cells migrate towards the stiffer side of the gradient. Created in BioRender by Pethö, Z., 2025. https://BioRender.com/g0g0gfu. This figure was sublicensed under CC-BY 4.0 terms. (H) Durotaxis polar plots depict individual PSC trajectories over 24 h (black lines) for stiff and soft areas. For each plot, the gradient orientation is towards the left side. The radii of the blue half circles on the right-hand and left-hand sides of each plot are proportional to the mean cellular displacement towards 0° and 180°, respectively. Radial lines indicate 0°, 90°, 180° and 270°. Scale bar: 100 µm for the migration trajectories and 50 µm for the half circles. Radius of the concentric gray circle is a visual aid for the scale bar. (I–L) Scatter plots show the PSC durotaxis index (I), velocity (J), displacement along the *X*-axis up the stiffness gradient (K) and area (L), on stiff (left) and soft (right) areas of the gradient hydrogels. *n*=56 cells measured across *N*=4 experiments. Data in B,C,F and I–L: median±95% confidence interval. Statistical tests: Mann–Whitney *U*-test in C,F and J–L; one-sample Wilcoxon test in I. a.u., arbitrary units; n.s., not significant.

Next, we assessed how PSCs perceive spatial differences in substrate stiffness representing the soft, healthy pancreatic tissue (1–2 kPa) and stiff tumorous tissue (5–10 kPa). Therefore, we established a gradient hydrogel system recapitulating both stiffnesses. We validated the substrate stiffness using AFM (Fig. 2A–C). AFM measurements showed a linear decrease in the stiffness of ECM-coated gels by ∼5 kPa over a distance of 2 mm in the region of the gradient (Fig. 2B). The stiffest part of the gradient hydrogel was in the upper range of the stiffness values of PDAC, whereas the softest area of the gel was in the upper range of the physiological stiffness of the healthy pancreas (Rubiano et al., 2018; Shi et al., 2018). The gel areas where the photomask was transparent (positions −5 mm to +1 mm) had a uniform stiffness, further underlying the specificity of UV polymerization with a photomask. To exclude that ECM coating affects hydrogel properties, we systematically investigated the gradient stiffness of the same gels before and after ECM coating (Fig. 2B), as well as before and after the 24 h cell migration assays (Fig. 2C). Importantly, the stiffness gradient remained stable throughout the whole experimental period.

When seeded onto hydrogels with a stiffness gradient, we observed that PSC morphology was different in stiff and soft areas of the gradient (Fig. 2D), reminiscent of PSC morphology on stiff and soft hydrogels (Fig. 1A). Next, we assessed the myofibroblastic phenotype of PSCs based on total cellular αSMA (also known as ACTA2) (Pethő et al., 2023), derived from αSMA fluorescence intensity multiplied by cell area (Fig. 2E,F). PSCs had more αSMA on the stiffer part of the gradient than on the softer part. Also, cell area and αSMA fluorescence intensity significantly differed between stiff and soft regions (Fig. S2A,B).

Next, we monitored the migratory behavior of PSCs over 24 h on the ECM-coated gradient hydrogels (Fig. 2G–L). We visualized the functional mechanosensitivity of PSCs using polar plots (Fig. 2H) and assessed the durotaxis index as a readout of the relative migration towards the stiff substrate (Fig. 2I). PSCs had a positive durotaxis index on both the stiffer and the softer halves of the gradient. Notably, on the stiffer part of the gel, cells generally migrated faster (Fig. 2J), leading to an increased net displacement along the stiffness gradient (*X*-axis) (Fig. 2K). Moreover, PSCs had a larger area (Fig. 2L) on the stiffer regions of the hydrogel. These results are consistent with the observations on the homogeneous gels (Fig. 1C–E): higher stiffness is associated with increased cell area and velocity.

### PSC durotaxis depends on Piezo1 channel activity

**Fig. 3.**
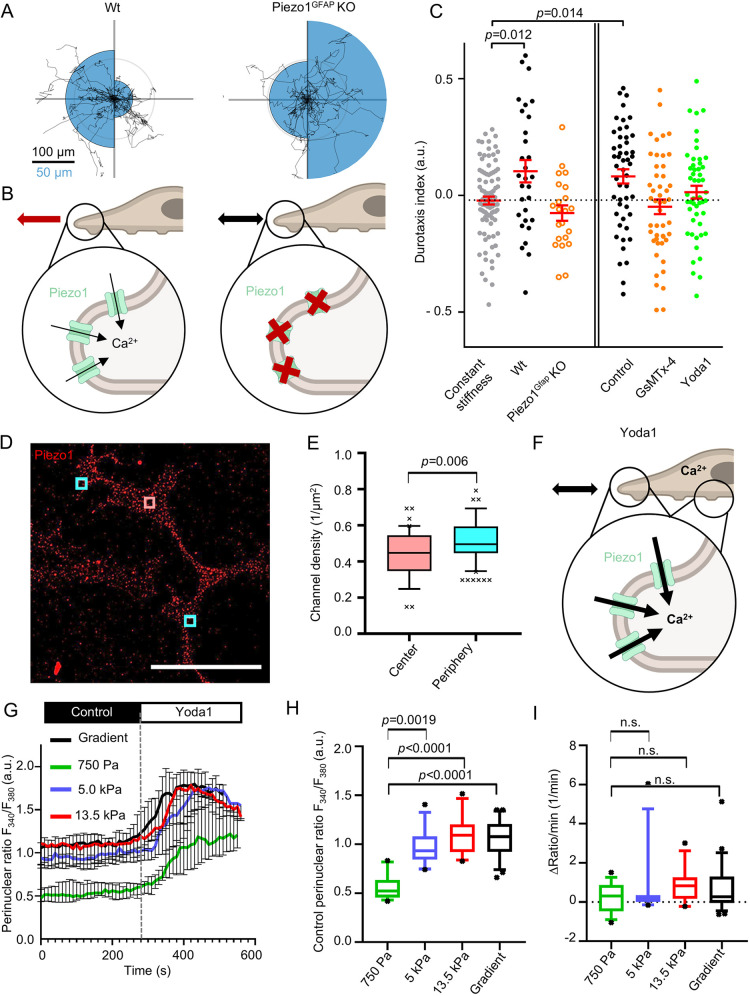
**Piezo1 is involved in PSC durotaxis.** (A) Durotaxis polar plots of wild-type (Wt, left) and Piezo1^GFAP^ KO PSCs depict cell trajectories over 24 h (black lines). For each plot, the gradient orientation is towards the left side. The radii of the blue half circles on the right-hand and left-hand sides of each plot are proportional to the mean cellular displacement towards 0° and 180°, respectively. Radial lines indicate 0°, 90°, 180° and 270°. Scale bar: 100 µm for the migration trajectories and 50 µm for the half circles. Radius of the concentric gray circle is a visual aid for the scale bar. *n*=29 cells for Wt, *n*=22 cells for Piezo1^GFAP^ KO from *N*=3 experiments. (B) Interpretation of results depicted in A. In Piezo1^GFAP^ KO PSCs the intracellular Ca^2+^ signal is missing in response to stiffness sensing. Thus, Piezo1^GFAP^ KO PSCs fail to perform durotaxis. (C) Durotaxis indices of Piezo1^GFAP^ KO PSCs (orange, open), PSCs treated with 100 nM GsMTx-4 (orange, filled), PSCs treated with 5 µM Yoda1 (green) and their respective controls (black) on stiffness gradient hydrogels, compared to PSCs on gels with constant stiffness (gray, mean marked by dotted line). Where cells undergo durotaxis, the *P* value is indicated. *n*≥22 cells from *N*≥3 experiments. (D) Representative immunofluorescence image of PSCs stained for Piezo1 (red), from *N*=3 experiments. Channels were quantified in rectangular regions in the cell center (red) and periphery (cyan). Scale bar: 50 µm. (E) Box and whisker plot shows quantification of Piezo1 channel density in rectangular regions as outlined in D. Center *n*=41, periphery *n*=82 from *N*=3 experiments. (F) Schematic interpretation of how Yoda1 treatment affects durotaxis by clamping Piezo1 activity to its maximal value throughout the cell. [Ca^2+^]_i_ increases over the whole cell, diminishing the differential stiffness sensing by Piezo1. (G) F_340_/F_380_ ratios were used as a readout for intracellular Ca^2+^ measurements of PSCs. The F_340_/F_380_ ratio is a surrogate of the [Ca^2+^]_i_. After control superfusion (black bar), PSCs seeded on hydrogels with varying stiffness (750 Pa, 5 kPa, 13.5 kPa, and gradient gels) were superfused with 5 µM Yoda1 (white bar). *n*≥13 cells from *N*=3 experiments. Gray vertical dashed line indicates the start of Yoda1 superfusion. (H) Box and whisker plots show quantification of F_340_/F_380_ ratios of PSCs superfused with control solution from experiments detailed in G. (I) Box and whisker plots indicate the slope of F_340_/F_380_ ratio upon Yoda1 application, from experiments detailed in G. Data in C are represented as mean±s.e.m., and G are median±95% confidence interval. Boxes in E, H and I extend from the 25th to 75th percentiles, whiskers from 10th to 90th percentiles, and points below and above the whiskers are drawn as individual points. Statistical tests are as follows: Dunnett's multiple comparison test in C; Mann–Whitney *U*-test in E, and Kruskal–Wallis test with Dunn's post-hoc test in H and I. Statistical comparison of panel C is further detailed in Table S2. a.u., arbitrary units; n.s., not significant. Images in B and F were created in BioRender by Pethö, Z., 2025. https://BioRender.com/szyhl8v. This figure was sublicensed under CC-BY 4.0 terms.

Having demonstrated that PSCs exhibit a stiffness-guided migratory behavior, the following sections focus on better understanding the molecular mechanisms behind the process of durotaxis. Mechanically evoked transient Ca^2+^ spikes elicited by ion channel activity at the anterior cell pole of migrating cells are known to control the directionality of cell movement (Wei et al., 2009; Holt et al., 2021). With this in mind, we performed a proof-of-principle experiment to test whether the mechanosensitive ion channel Piezo1 is involved in durotaxis. We employed a genetic model and used PSCs from wild-type (Piezo1^fl/fl^, also referred to here as Piezo1^WT^) mice or from mice that harbor a glial fibrillary acidic protein (GFAP)-dependent conditional Piezo1 KO (Piezo1^GFAP^ KO) (Fig. 3A). The Piezo1^GFAP^ KO PSCs elicited no Ca^2+^ signal upon stimulation with the Piezo1 activator Yoda1 (Fig. S3) (Romac et al., 2018; Swain et al., 2022). Indeed, Piezo1^GFAP^ KO PSCs did not perform durotaxis on ECM-coated gradient hydrogels, compared to control PSCs on gels with uniform stiffness (Fig. 3B). Pharmacological modulation of Piezo1 – by either inhibiting the channel with 100 nM GsMTx-4 or activating the channel using 5 µM Yoda1 – further corroborated its role in durotaxis: (Fig. 3C; Fig. S4). Whereas the control PSCs, treated with 0.1% DMSO, were found to undergo durotaxis, both GsMTx-4- and Yoda1-treated PSCs had a durotaxis index not significantly different from 0 (corresponding to random migration; Table S2). The data from GsMTx-4-treated PSCs and Piezo1^GFAP^ KO PSCs indicate that the ability of PSCs to dynamically adapt their Piezo1 activity to environmental cues is essential for durotaxis (Fig. 3C; Table S3).

We followed up on investigating why Piezo1 activation with Yoda1 diminishes durotaxis (Fig. 3C). One possibility is that spatially different substrate stiffness signals to the cell by localized Piezo1 activity. Alternatively, inhomogeneous channel expression could transmit differences in substrate stiffness. To address the heterogeneity of Piezo1 in the plasma membrane of PSCs, we performed Piezo1 immunocytochemistry on the gradient hydrogels and analyzed the spatial channel distribution. Channel density was quantified in 5 µm^2^ regions in the cell center or cell periphery (Fig. 3D). As Fig. 3E illustrates, the average channel density was ∼0.5 channels/µm^2^, with the cell periphery having a marginally higher channel density than the cell center. This would imply that global Piezo1 activation by Yoda1 elicits similar Ca^2+^ signals throughout the cell that are insufficient for spatial stiffness sensing (Fig. 3F). Measurements of the intracellular Ca^2+^ concentration, [Ca^2+^]_i_ using the ratiometric Ca^2+^ indicator dye Fura-2 revealed that the initial F_340_/F_380_ ratio (the ratio of fluorescence intensity at 340 nm excitation and 380 nm excitation) was significantly lower in PSCs on a 750 Pa substrate compared to that in cells on stiffer substrates (Fig. 3G,H). However, upon superfusing the PSCs with 5 µM Yoda1, the F_340_/F_380_ ratio increased similarly across all substrates, implying a full activation of Piezo1 throughout the plasma membrane (Fig. 3I). These results reinforce the hypothesis that uniform Piezo1 activation overrides spatial stiffness cues, impairing durotaxis.

### Piezo1 orchestrates PSC durotaxis together with TRPV4 and TRPC1

There is strong evidence that Piezo1 executes cellular functions by acting in conjunction with another ion channel, TRPV4, which is known as a signal amplifier for pressure sensing (Swain et al., 2020). Thus, we next investigated whether TRPV4 is involved in PSC durotaxis. We applied either a pharmacological TRPV4 activator (GSK1016790A; 100 nM) or the TRPV4 inhibitor HC067047 (100 nM). Additionally, we investigated whether the TRPC1 channel mediates durotaxis, as this channel is required for directional migration in response to chemical and mechanical cues (Fabian et al., 2008, 2012), and we have previously found TRPC1 to regulate pressure-mediated PSC activation (Radoslavova et al., 2022). For this, we used PSCs isolated from TRPC1 KO mice. TRPC1 KO PSCs show unaltered *Piezo1* expression but upregulate *TRPV4* expression (Fels et al., 2016).

**Fig. 4.**
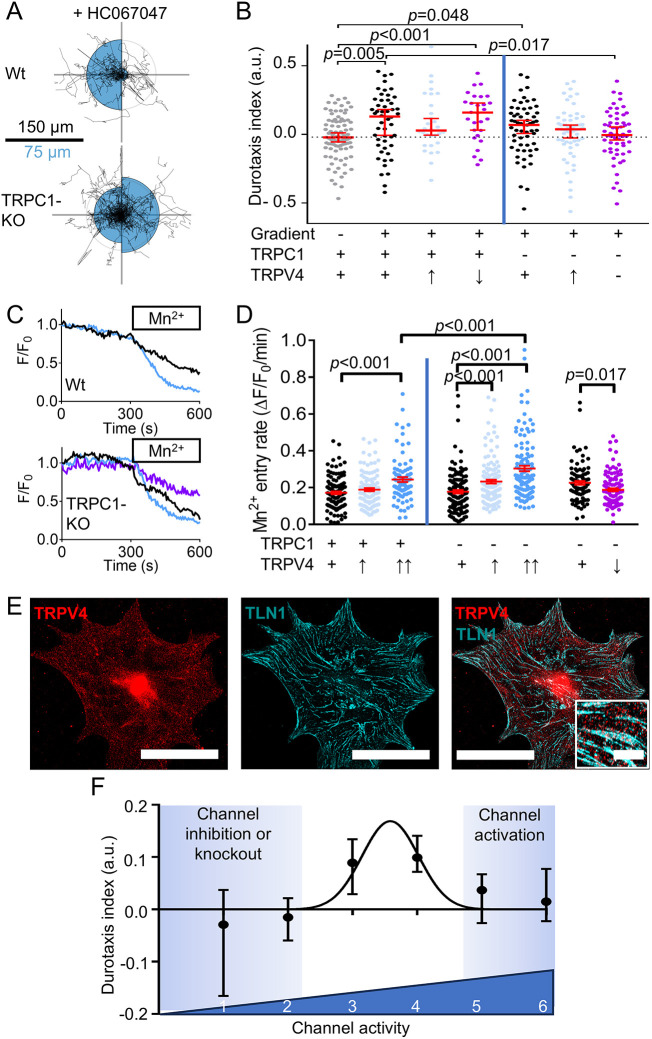
**Multiple mechanosensitive channels are necessary for durotaxis.** (A) Durotaxis polar plots depict cell trajectories over 24 h (black lines) of wild-type (Wt, top) and TRPC1 KO (TRPC1-KO, bottom) PSCs treated with the TRPV4 inhibitor 100 nM HC067047. For each plot, the gradient orientation is towards the left side. The radii of the blue half circles on the right-hand and left-hand sides of each plot are proportional to the mean cellular displacement towards 0° and 180°, respectively. Radial lines indicate 0°, 90°, 180° and 270°. Scale bar: 100 µm for the migration trajectories and 50 µm for the half circles. Radius of the concentric gray circle is a visual aid for the scale bar. *n*=28 for Wt and *n*=59 for TRPC1-KO cell trajectories from *N*=3 experiments. (B) Durotaxis indices of *n*=28 wild-type PSCs (TRPC1 +) from *N*=3 experiments and *n*=59 PSCs from TRPC1 KO mice (TRPC1 −) from *N*=3 experiments (left and right from vertical line, respectively) seeded on gradient hydrogels treated with 0.1% DMSO (black), 10 µM TRPV4 activator GSK1016790A (TRPV4 ↑, light blue) or 100 nM HC067047 (TRPV4 ↓, purple). They are compared with wild-type PSCs seeded on gels with homogeneous stiffness (gray, median marked by dotted line). *n*=50 cell trajectories from *N*=4 experiments. (C) Representative graphs of Mn^2+^ quench experiments showing the relative fluorescence intensity (F/F_0_) of Fura-2 AM-loaded wild-type and TRPC1 KO PSCs. Upon application of Mn^2+^ (box), quenching of the Fura-2 signal can be observed under control conditions (black), in the presence of 20 nM GSK1016790A (blue) or 2 µM HC067047 (purple). (D) Mn^2+^ entry rates of wild-type PSCs (TRPC1 +) and PSCs from TRPC1 KO mice (TRPC1 −) determined under control conditions (TRPV4 +) and in the presence of GSK1016790A (20 nM, TRPV4 ↑; 100 nM, TRPV4 ↑↑) or 2 µM HC067047 (TRPV4 ↓), as indicated. *n*≥87 cells from *N*=3 experiments. (E) Representative immunofluorescence image of a PSC stained for TRPV4 (red) and TLN1 (cyan), with magnified image in the inset. Scale bars: 50 µm; inset, 5 µm. Images are representative of *N*=3 experiments. (F) Durotaxis indices as a function of ion channel activity. To estimate total channel activity, individual channel activity was binned: impaired channel=0; intermediate activity=1 for TRPV4 and TRPC1; intermediate activity=2 for Piezo1; overactivation=3 for TRPV4; and overactivation=4 for Piezo1. A Gaussian curve was fitted over the data. Data in B are represented as median±95% confidence interval; data in D are represented as mean±s.e.m.; data in F are represented as median±95% confidence interval. Statistical tests: Kruskal–Wallis statistical test with Dunn's post-hoc test in B; one-way ANOVA followed by Tukey's post-hoc test in D. Statistical comparison of panel B is further detailed in Table S2. a.u., arbitrary units.

We found that pharmacological activation of TRPV4 impaired durotaxis in PSCs isolated from wild-type and TRPC1 KO mice (Fig. 4A; Fig. S5). This effect is similar to the diminished durotaxis observed upon Piezo1 activation with 5 µM Yoda1 (Fig. 3C; Fig. S4). In contrast, neither TRPV4 inhibition in wild-type PSCs nor TRPC1 KO alone affected durotaxis. However, inhibiting TRPV4 in PSCs from TRPC1 KO mice hampered durotaxis, indicating that Piezo1 channels by themselves are not sufficient to orchestrate durotaxis but require the cooperative activity of TRPV4 and/or TRPC1 (Fig. 4B).

We assumed that the observed effects of TRPV4 and TRPC1 modulation of durotaxis are due to the Ca^2+^ influx mediated by these channels. To explore how channel activity and Ca^2+^ influx are affected by TRPV4 and TRPC1 modulation, we next performed a set of Mn^2+^ quench experiments on PSCs (Fig. 4C). Here, the Mn^2+^ entry rate acts as a surrogate of the Ca^2+^ influx into the PSCs. As observable from Fig. 4D, the TRPV4 activator GSK1016790A acted in a dose-dependent manner on PSCs: in wild-type PSCs 100 nM but not 20 nM, GSK1016790A elicited a marked Ca^2+^ influx. Moreover, TRPV4 modulation led to more pronounced effects in TRPC1 KO PSCs as compared to wild-type PSCs. In TRPC1 KO PSCs, 20 nM GSK1016790A was sufficient to increase the Ca^2+^ influx rate, and 100 nM GSK1016790A significantly further amplified Ca^2+^ influx as compared to the influx observed for wild-type PSCs. We also tested whether diminished durotaxis upon TRPV4 inhibition in TRPC1 KO PSCs is due to impaired Ca^2+^ influx. Indeed, we found a decreased Ca^2+^ influx rate in the presence of the TRPV4 inhibitor HC067047 (100 nM) versus the control. The finding that durotaxis under combined TRPC1 KO and TRPV4 inhibition is diminished compared to control WT PSCs emphasizes that TRPC1 and TRPV4 are relevant in regulating PSC durotaxis.

To gain insights into the cellular distribution, we visualized TRPV4 in comparison to the focal adhesion protein Talin-1 (TLN1) via immunocytochemistry (Fig. 4E). We observed that TRPV4, similar to Piezo1 (Fig. 3D), was universally distributed throughout the PSCs, whereas Talin-1 showed marked accumulations, plausibly in focal adhesion sites. Some of these sites also overlapped with TRPV4 signals, implying they might contain TRPV4 channels. The results above imply that durotaxis depends in a bell-shaped manner on ion channel activity. Both the fixed loss of the Piezo1 channel function by Piezo1^GFAP^ KO or GsMTx-4 treatment and fixed gain of channel function by Yoda1 treatment resulted in loss of the ability to migrate towards stiffer areas. We found a similar phenomenon upon TRPV4 activation and inhibition, especially when combined with TRPC1 KO. We next developed a conceptual framework of the bell-shaped dependence of durotaxis on ion channel activity (Fig. 4F). In the control state, the intermediate relative activities of TRPV4 and TRPC1 are illustrated by an arbitrary value of 1. Because of the paramount functional relevance of Piezo1 in PSC durotaxis, its activity was assigned an intermediate value of 2. Inhibition or KO of TRPV4, TRPC1 and Piezo1 channels correspond to a value of 0. Pharmacological activation of TRPV4 and Piezo1 channels is represented by the values 3 and 4, respectively. The application of these values results in a score for the combined activity of these ion channels with values between 2 and 6: a value of 2 indicates GsMTx-4 treatment, Piezo1 KO, or dual TRPC1 KO and TRPV4 inhibition when channel activity is substantially impaired; a value of 5 or 6 corresponds to a state when TRPV4 and Piezo1 are pharmacologically clamped in an overactivated state (Fig. 4F; detailed in Fig. S6 and Table S3). Our results emphasize that the three channels need to have an intermediate level of activity (value of 3-4) for optimal durotaxis. Whenever the dynamics of channel activity are substantially affected, either by tonic inhibition or overactivation, the cell will not be able to sense (and thus react to) changes in local substrate stiffness. Notably, an intermediate activity of Piezo1 is necessary, but not sufficient for durotaxis. Efficient durotaxis of PSCs requires the additional function of TRPV4 or TRPC1.

### A durotaxis model with ion channels and a bell-shaped mechanosensitivity function

Above, we outlined the hypothesis that an intermediary combined activity of Piezo1, TRPC1 and TRPV4 is necessary for durotaxis. Here, we support and refine this hypothesis with a mathematical model consisting of a system of partial differential equations. Mathematical models of durotaxis have been published previously (Allena et al., 2016; Malik and Gerlee, 2019). In Allena et al. (2016), a cellular Potts model is analyzed for a single cell moving over a flat substrate with variable stiffness. The cell is described by several lattice sites. Cell dynamics result from an iterative stochastic reduction of the energy of the whole system, and a modified Metropolis method for Monte Carlo–Boltzmann dynamics is employed. In Malik and Gerlee (2019), a stochastic model is introduced where the cell moves by updating its adhesion sites at random times. The rate of updating is determined by the local stiffness of the substrate. From this, a model of partial differential equations of advection–diffusion type is formally derived, where the advective velocity relates to the stiffness, like in the mathematical model we suggest below. In our case, however, we additionally couple to channel dynamics. The advection–diffusion equation in Malik and Gerlee (2019) is numerically solved by a Crank–Nicolson finite difference scheme. How to formally obtain advection terms for taxis terms like in Malik and Gerlee (2019) is also discussed in Stevens and Othmer (1997).

For our mathematical model, *u=u(x,t)* denotes the probability density of the cell in space and time, and *ρ*=*ρ*(*x*, *t*) and *η*=*η*(*x*, *t*) denote the densities of the open and closed channels of the cell, respectively. The time–space dynamics of the cell depend on the relative density 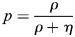 of open channels and is given by:
(1)

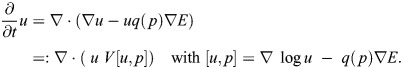


**Fig. 5.**
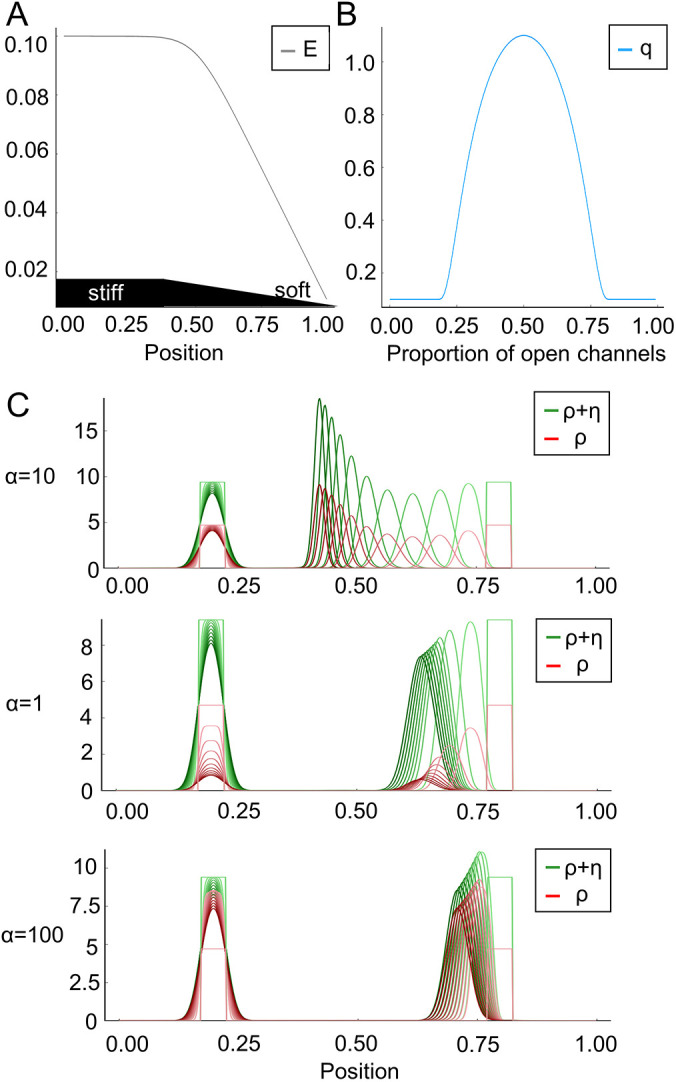
**The mathematical model supports the hypothesis of the dependence of durotaxis on ion channel function.** (A) *E* denotes the elastic modulus of the substratum; it is larger for a stiffer material. The substratum's stiffness gradient is indicated in black. (B) The bell-shaped function *q* accounts for the strength of the cell to sense the gradient of *E* due to the relative number of open versus closed channels. (C) The diagram depicts the velocity of the probability density of the cell (green) in dependence of the channel opening rate *α*. Red is the density of open channels. *α*=10 corresponds to an intermediate channel activity, whereas *α*=1 and *α*=100 indicate an impaired or overactivated channel function, respectively. The closing rate is *β*=1. For a comparison, initially two cells (green rectangles) are located at around 0.2 (stiffer region) and 0.8 (less stiff region). The cell located on the right moves from right to left, towards the stiffer region. The cell on the left is already located in the stiffer region and hardly changes position at all. Initial time points are shown in light green and red, with colors becoming progressively darker for subsequent time points. These snapshots are equidistant in time. PSC movement towards the stiffer region is attenuated if the mechanosensitive channels are closed (α=1) or overactivated (α=100).

In Eqn 1, the first term on the right-hand side accounts for random motion of the cell, and the second term for its tendency to move into the direction of higher stiffness of the substratum. The second line of the formula is a reformulation convenient for the numerics (see details in the Numerics section of the Materials and Methods). Here *E* denotes the elastic modulus of the substratum, which has a larger value for a stiffer material. The function *q* accounts for the strength of the cell to sense the gradient of *E* due to the relative number of open versus closed channels *p*. It is assumed to be bell shaped – as suggested by data in Fig. 4 – with a maximum at *p*=0.5 (i.e. *ρ*=*η*) and minimal values at *p*=0, *p*=1 (i.e. *ρ*=0, *η*=1and *ρ*=1, *η*=0). See Fig. 5A,B for the one-dimensional situation on the space interval [0,1]. The dynamics of open and closed channels are given by:
(2)

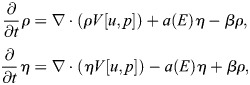
that is, the channels are located on the cell and thus move with the cell. Further, *a*=*a*(*E*) is the opening rate of the channels, which depends on the elastic modulus *E*, and the constant *β* is the closing rate. All partial differential equations are analyzed on a bounded domain with no-flux boundary conditions. The mass of *u* and of *ρ*+*η* is conserved. Furthermore, if initially (i.e. at time *t*=0) *u*(0, *x*)=*C*(*ρ*(0, *x*)+*η*(0, *x*)) for some constant C>0, then this feature will be preserved, that is, *u*(*t*, *x*)=*C*(*ρ*(*t*, *x*)+*η*(*t*, *x*)) for all *t*>0. This means that any chosen total initial distribution of channels in the model is preserved over time. In particular we have *V*[*u*, *p*]=*V*[*ρ*+*η*, *p*] in Eqn 1 due to the logarithm and the gradient. Once the constant C is fixed, it is sufficient to solve system Eqn 2, which can be reformulated in the self-consistent form:
(3rma)

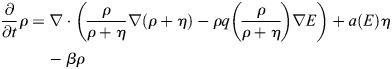

(3rmb)

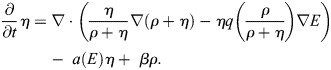


Now we set the opening rate of the channels proportional to the elastic modulus: *a*(*E*)=*αE* for *α*>0. The closing rate *β* is independent of *E*. We consider a one-dimensional spatial setting in the numerical simulations of this mathematical model. Without loss of generality, the strength of random motion is fixed to one in Eqn 1. When *α*=10, the simulation results in intermediate channel activity (Fig. 5C) and recapitulates that cells persist towards the stiffer substrate. Intriguingly, during movement towards the stiffer substrate, the density of open channels increases over time. In contrast, the condition *α*=1 recapitulates GsMTx-4 treatment, Piezo1^GFAP^ KO, or TRPC1 KO together with TRPV4 inhibition, where ion channel function is diminished. Here, impaired channel activity further hampers durotaxis on stiffer substrates over the course of the simulation. A similar inhibition of durotaxis is apparent in the condition *α*=100, which corresponds to channel overactivation as in the presence of Yoda1. Taken together, these simulations show that PSC durotaxis can be effectively modeled via a bell-shaped relationship between ion channel activity and durotaxis.

## DISCUSSION

In this study, we provide a mechanistic link between ion channels and durotaxis of PSCs. We demonstrate that PSC durotaxis is diminished by perturbing ion channels involved in mechanosensing and mechanosignaling (Figs 3, 4). Our key finding is that Piezo1 is essential for the durotaxis of PSCs (Fig. 3) but requires functional cooperation with TRPC1 and TRPV4 channels (Fig. 4). Both overstimulation and inhibition of these ion channels reduce durotaxis. Our mathematical model supports the conclusion that durotaxis of PSCs is optimal at intermediate levels of dynamically fluctuating activity of ion channels (Fig. 5). Stated differently, durotaxis depends on ion channel activity in a bell-shaped manner. This bell-shaped relationship would explain how channel inhibition or excessive pharmacological activation, such as Piezo1 activation using Yoda1 (Syeda et al., 2015) as well as that of TRPV4 with GSK1016790A (Thorneloe et al., 2008), lead to altered Ca^2+^ influx and impair durotaxis (Figs 3, 4). As a consequence of inappropriate spatiotemporal control of Ca^2+^-permeable channel activity, Ca^2+^ concentrations diverge from the optimum and hamper durotaxis, analogous to findings in oxidative phosphorylation (Vilas-Boas et al., 2023). In addition, similar bell-shaped relations between migratory behavior and ion transport are also known for other transport proteins such as the Na^+^–H^+^ exchanger (Stock et al., 2005) and K_Ca_3.1 channels (Schwab et al., 1999). Our mathematical model also indicates that PSCs will accumulate on the stiffer substrate over time, suggesting that PSCs will remain in stiff tumor tissue. Hence, durotaxis regulation by Piezo1, TRPV4 and TRPC1 channels constitutes a feed-forward mechanism contributing to PSC accumulation and consequent PDAC fibrosis. This adds to the mechanical activation that is also modulated by ion channels involved in mechanosignaling, such as TRPC1 (Radoslavova et al., 2022).

One strength of our study is the use of pathophysiologically relevant substrate rigidities and gradients. By using a two-dimensional stiffness gradient in our biological system, we were able to monitor every gradient hydrogel with AFM. We found that UV polymerization using a photomask produced a reproducible, linear stiffness gradient of 2.5 kPa/mm (Fig. 2B). The length of a PSC is ∼50–100 µm, depending on the underlying substrate. This means that a PSC can sense a gradient of less than 250 Pa between the cell rear and front, on a substrate having a 2–8 kPa stiffness, highlighting the sensitivity of the mechanosensitive cellular network. The durotaxis index is similar on the soft and the stiff part of the gradient gel (Fig. 2I), indicating that the directed and undirected proportion of the cell migration are more or less the same, although cells migrate with a different velocity along the stiffness gradient (Fig. 2J,K). This is reflected in the one-dimensional mathematical model by the specific choice of the channel opening rate being proportional to *E*. It likely indicates that the stiffness difference between cell rear and front is more important for durotaxis than the absolute stiffness value. Further studies will outline how these results are applicable to a confined, three-dimensional PDAC tissue.

Our study focuses on substrate stiffness and does not address the viscous properties of the underlying substrate. Elevated viscosity induces a TRPV4-mediated mechanical memory through transcriptional regulation via the YAP–TAZ pathway (Bera et al., 2022). Indeed, YAP1 undergoes increased nuclear translocation in PSCs on a stiff substrate compared to that in PSCs on a soft substrate, resulting in a more myofibroblastic phenotype and increased αSMA expression (Pethő et al., 2023). Hence, it is likely that the tissue viscosity plays an additional role in PSC migration and durotaxis in our experimental system and even more so in the complex PDAC tissue.

Our data suggest that durotaxis in PSCs relies on the localized activity of ion channels, which we summarized in a conceptual model (Fig. 4F). In this model, the activity of the Ca^2+^-permeable channels Piezo1, TRPC1 and TRPV4 (*X*-axis) leads to local fluctuations of the intracellular Ca^2+^ concentration. These fluctuations are hypothesized to influence durotaxis indices (*Y*-axis), potentially by differentially modulating focal adhesion dynamics at cell poles oriented towards stiffer versus softer substrate regions. Furthermore, the intracellular Ca^2+^ concentration of PSCs is influenced by the stiffness of their surrounding ECM. In light of the fact that Piezo1 and TRPV4 have enhanced activity in the vicinity of focal adhesions (Ellefsen et al., 2019; Matthews et al., 2010), a local channel activation is expected in the cell regions adjacent to the stiffer matrix regions of the gradient gel. Also, Piezo1 binds to matrix adhesions in a force-dependent manner in non-cancerous cells, promoting adhesion dynamics and Ca^2+^ entry (Yao et al., 2022). Accordingly, we have found previously that Piezo1-mediated Ca^2+^ influx into PSCs is hampered upon impairment of myosin function using blebbistatin (Kuntze et al., 2020). These findings suggest that Piezo1-mediated Ca^2+^ signaling in PSCs is more pronounced in regions of high contractile forces, aligning with the stiffness gradient. Nevertheless, the question of whether Piezo1 activity scales directly with substrate stiffness during durotaxis remains open and warrants further investigation.

An uncoordinated activation of one of the three ion channels investigated in this study – for example, with the Piezo1 activator Yoda1 (Fig. 3G–I) or the TRPV4 activator GSK1016790A (Fig. 4B) – would likely disrupt spatiotemporal Ca^2+^ signaling and impair cell polarization and durotaxis. In our system, we could not experimentally test this due to technical limitations, namely the thickness and optical properties of the coated polyacrylamide gel. However, it can be assumed that PSCs, like other fibroblasts, actively detect the strength of the surrounding ECM by traction (Sun, 2021). If they are subjected to a stiffness gradient, traction on the stiffer regions will induce less substrate deformation. This lower compliance would result in a higher membrane tension of the lipid membrane of the cell, which in turn could be detected by the Piezo1 channel. According to the force-from-lipid model, this would provide a suitable stimulus for opening the channel (Kobayashi and Sokabe, 2010; Syeda et al., 2016; Martinac et al., 1990). This would ultimately lead to a local Piezo1 and subsequent TRPV4 activation and a regional influx of Ca^2+^ into the cell, resulting in a stronger pulling than on the soft part of the cell.

Piezo1 is known to act together with other TRP channels: in response to pressure overload, Piezo1 transduces mechanical signals to TRPM4 channels in cardiac hypertrophy (Yu et al., 2022), and Piezo1 cooperates with TRPV4 channels in pressure-induced pancreatitis (Swain et al., 2022). Similarly, during durotaxis, primary mechanosensing could occur through Piezo1 but be translated into a sufficient cellular response only by subsequent activation of TRPV4. A possible mechanism would be similar to that in endothelial cells, where TRPV4 responds to lipid signaling products generated by Piezo1 activation (Swain and Liddle, 2021). Along these lines, TRPC1 likely perpetuates pressure-mediated PSC activation: TRPC1 is linked to Ca²^+^-dependent activation of intracellular signaling cascades, such as the Erk1/2 and Smad2 pathways in PSCs, which are known to regulate key cellular functions like αSMA expression, stiffness modulation and IL-6 secretion (Radoslavova et al., 2022). Thus, we assume that TRPC1 and TRPV4 act as downstream effectors and amplifiers of mechanical stimuli, as the intrinsic mechanosensitivity of TRPC1 and TRPV4 has not been clearly demonstrated (Nikolaev et al., 2019; Maroto et al., 2005; Gottlieb et al., 2008). Moreover, the functional expression of TRPV4 is linked to the presence of TRPC1: TRPV4 is functionally upregulated in TRPC1 KO PSCs, as demonstrated by our Mn^2+^ quench experiments (Fig. 4D). This is well in line with our previous findings revealing increased *TRPV4* mRNA expression in TRPC1 KO PSCs (Fels et al., 2016).

We argue that the functional interplay between Piezo1, TRPV4 and TRPC1 might have a pathophysiological relevance in PDAC and other tumor entities. A major driver of PDAC progression is the dense, collagen-rich stroma (Pothula et al., 2020). For other tumors, a correlation between elevated tissue stiffness, increased mechanosignaling and tumor malignancy could be demonstrated (Acerbi et al., 2015). Analogously, for PDAC, a positive mechanical feedback loop involving stiffness-guided activation and migration of PSCs has been postulated (Lachowski et al., 2017; Pethő et al., 2019), in which Piezo1 would act as the primary sensor of pathologically increased environmental stiffness. Initial signals would then be amplified by other ion channels, such as TRPV4 and TRPC1, followed by a change in cell phenotype, potentially inducing myofibroblastic differentiation. Myofibroblastic PSCs would then recruit additional cells and secrete an abundance of ECM proteins, culminating in a fibrotic PDAC stroma. Indeed, PSCs spread more on substrates with higher stiffness, leading to a more effective cell migration with a higher velocity (Fig. 1). When introducing a stiffness gradient, PSCs migrate towards a higher stiffness, where they acquire increased levels of αSMA (Fig. 2). The morphological transition and increase in αSMA point towards an increase in the myofibroblastic machinery, which can explain the higher migration velocity on the stiffer part of the gradient hydrogel (Fig. 2J). However, whether impairment of this mechanical feedback loop can be therapeutically targeted *in vivo* in PDAC fibrosis remains to be investigated. In summary, PSC durotaxis relies on the interaction between multiple ion channels that cooperate with mechanosensitive Piezo1 channels. These results support the idea that the consequences of durotaxis should be further explored in diseases such as PDAC to better understand tumor fibrosis and progression.

## MATERIALS AND METHODS

### Laboratory animals

The isolation of stellate cells from mouse pancreas has been reported to the Landesamt für Natur, Umwelt und Verbraucherschutz Nordrhein-Westfalen (LANUV) in Recklinghausen under the following number: 84-02.05.50.15.010. If not specified otherwise, 8- to 15-week-old wild-type mice (C57 BL/6J) were used for the experiments. In addition, we used PSCs from mice with a global TRPC1 KO and conditional stellate cell-specific Piezo1 KO (Piezo1^GFAP^ KO) (Dietrich et al., 2007; Swain et al., 2022). Here, the *GFAP* gene was used as a marker gene to identify and target PSCs for the conditional KO of Piezo1. PSC isolation from Piezo1^GFAP^ KO mice was performed with approval from the Institutional Animal Care and Use Committee and Institutional Review Board of Duke University (protocol number Pro00035974), and isolation from TRPC1 KO mice was approved by local animal welfare committee (LANUV).

### Cells and culture conditions

PSCs were isolated from the pancreas as described previously (Kuntze et al., 2020). Briefly, after dissection of the pancreas, the organ was washed in GBSS (PAN Biotech, Germany), then cut into pieces and digested using collagenase P (1 mg/ml; Roche, Switzerland) in GBSS for 20 min at 37°C in a shaker. After washing and centrifugation at 300 ***g*** at room temperature for 8 min, the pelleted pancreas was reconstituted in DMEM/Ham's-F12 medium (PAN Biotech, Germany). The suspension was seeded dropwise onto fetal calf serum (FCS)-coated cell culture dishes (Corning, USA). After 2 h incubation at 37°C and 5% CO_2_, non-adherent cells were washed away by multiple washing steps. Cell removal was validated under the microscope using morphological observation. PSCs from Piezo1^GFAP^ KO mice were isolated in a comparable manner but were plated on a thin-layered Matrigel-coated glass-bottom culture plate (P35G-0-14-C, MatTek, USA) (Swain et al., 2022). PSCs in passage 1 were used for all subsequent experiments.

### Polyacryamide hydrogels

Uniform polyacrylamide gels were created using chemical polymerization (Rheinlaender et al., 2020). The polyacrylamide solution contained acrylamide (40%) (AppliChem GmbH, Germany), bisacrylamide (2%) (Carl Roth, Germany), hydroxyacrylamide (100%) (Sigma-Aldrich Chemie, Germany) and PBS. Gel stiffness was tuned by varying the concentrations of the polymer components (acrylamide, bisacrylamide and hydroxyacrylamide) (Table S1). The solution was ultrasonically degassed for 30 min and, thereafter, the polymerization was started by adding 0.003% N,N,N′,N′-tetramethylethylenediamine (TEMED) and 1% ammonium persulfate (APS) (Sigma-Aldrich Chemie, Germany). Next, 8 µl of the final polyacrylamide mix was pipetted onto 35 mm glass-bottom dishes (Cell E&G LLC VWR, USA) that were pretreated with 0.1 M NaOH, 200 µl APTMS (Sigma-Aldrich Chemie, Germany) and 0.5% glutaraldehyde (SERVA Electrophoresis, Germany). A RainX (ITW Global Brands, USA)-coated coverslip was lowered onto the solution and the glass-bottom dish was filled with PBS. The coverslips were left overnight and were then removed. The gels were then washed twice with sterile PBS and stored at 4°C.

For creating hydrogels containing a stiffness gradient, we irradiated a polyacrylamide solution containing the photoinitiator 2-hydroxy-4′-(2-hydroxyethoxy)-2-methylpropiophenone (Irgacure 2959) with UV light. We controlled the amount of UV light reaching the gel during the process of polymerization by placing a photomask with a linear opacity gradient (using Adobe Illustrator, Adobe, USA) between the light source and the gel mix. Thereby, the completeness of the gel formation and hence the stiffness were tuned. The protocol is based on the work of Sheth et al. and Tse and Engler (Sheth et al., 2017; Tse and Engler, 2010). The solution was ultrasonically degassed for 30 min, and afterwards the photoinitiator Irgacure 2959 was added. Then, 25 µl of the mix was transferred onto glass-bottom dishes pretreated with 0.1 M NaOH and silanized with 200 µl APTMS. A RainX-coated coverslip was lowered onto the solution, and dishes were then placed on the sample tray of the ChemiDoc Touch Imaging System (Bio-Rad, USA). The mask was placed between the dishes and the UV light source. After 720 s of illumination time the gels were immediately treated with PBS and the coverslips were removed.

### Atomic force microscopy

Polyacrylamide hydrogel stiffness and stiffness gradients were quantified using AFM. The Nanowizard 3 NanoOptics AFM system from Bruker Nano GmbH (Berlin, Germany) and the associated SPM software were used for this purpose. Only cantilevers from Novascan Technologies, Inc. (Boone, USA) with spherical tips and a tip diameter of 10 µm were used. According to the manufacturer, the spring constants were between 0.03 and 0.04 N/m and were determined at the beginning of the measurement using the thermal noise method (Cook et al., 2006). The sensitivity calibration of the cantilever was also performed at the beginning of each measurement. For this purpose, a test measurement was made on glass. The sensitivity of the cantilever was calculated from the slope of the linear section of the curve of the deflection signal. All measurements were performed at room temperature and in 2.5 ml sterile PBS. Gel stiffness was determined in 1 mm intervals along the *X* and *Y* axes. This ensured that the desired stiffness gradient was present in the 2×2 mm area selected for the subsequent tests. Five force–distance curves were generated for each measurement position. At 24 h after hydrogel coating (see below), the stiffness was measured at ten further points to verify gradient integrity.

### Hydrogel coating and cell seeding

All hydrogels were coated with a collagen matrix resembling the ECM of the pancreas, containing laminin, fibronectin, and collagens I, III and IV, as previously described (Nielsen et al., 2017; Kuntze et al., 2020). ECM proteins were covalently linked to the gel using sulfosuccinimidyl-6-(4'-azido-2'-nitrophenylamino)hexanoate (sulfo-SANPAH; Sigma-Aldrich Chemie, Germany), a photoreactive heterobifunctional crosslinker. After 15 min of irradiation with a wavelength of 302 nm, the gels were washed twice with sterile PBS. After repeating the irradiation and the washing process, 30 µl of the ECM premix was pipetted onto the gel. After a 2 h incubation period at 37°C, unbound matrix molecules were removed by washing with PBS.

### Measurement of cell migration

Cell migration time-lapse images were obtained using an Axiovert 40C phase-contrast microscope (Carl Zeiss AG, Germany) with an 10× objective (100× total magnification, Carl Zeiss AG, Germany). The acquisition was positioned on the 2×2 mm areas previously measured by AFM. Cell migration was recorded for 24 h at a time interval of 30 min.

Shortly before the start of the experiment, the DMEM/F12 medium was replaced by a 20 mM HEPES-buffered RPMI medium (Sigma-Aldrich). If necessary, the medium of the wild-type PSCs was supplemented with the Piezo1 activator Yoda1 (5 µM; Tocris), the inhibitor of cation-selective mechanosensitive ion channels GsMTx-4 (100 nM; Alomone, Israel), the TRPV4 agonist GSK1016790A (100 nM; Alomone, Israel) or the TRPV4 inhibitor HC067047 (100 nM; MedChemExpress, USA) (Syeda et al., 2015; Bae et al., 2011; Thorneloe et al., 2008; Everaerts et al., 2010). TRPC1 KO cells were also treated with the latter two channel modulators (GSK1016790A and HC067047). All listed substances were dissolved in DMSO, therefore 0.1% DMSO was added to the medium of the associated control experiments. Migration videos were analyzed using Amira-Avizo Software 2019.2 (Thermo Fisher Scientific, Waltham, MA, USA) and by using the MATLAB program (MathWorks, Inc., USA). Cell outlines were manually segmented in Amira. Subsequently, MATLAB was used to calculate the cell perimeter, cell centroid and cell area. From these data, we calculated further parameters. Cell circularity is quantified by the equation:

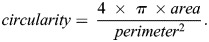
Cell movement parameters were velocity (µm/min; cell centroid displacement as function of time) and durotaxis index, which is defined by:

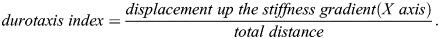
For depicting durotaxis, we developed durotaxis plots using MATLAB. For these plots, cell migration data were converted to polar coordinates and individual trajectories were visualized. To explore directional bias, the data were segmented into π radian increments, and the average magnitude of cell movement in each segment was calculated. These averages were then plotted as a qualitative polar histogram. The trajectories and histogram were then overlaid, offering a combined view of individual migration patterns and general directional tendencies.

### Immunofluorescence staining

For αSMA and vimentin immunostaining of PSCs, gradient gel-containing glass-bottom dishes were fixed with ice-cold methanol after the migration experiments. αSMA was used as a marker for cell activation (i.e. a myofibroblastic PSC phenotype), whereas vimentin was used as a marker to verify the quality of PSC isolation. Vimentin-positive PSCs were always the overwhelming majority of the cell population, as compared to vimentin-negative cells of epithelial origin and immune cells. For TRPV4 and Talin-1 immunostaining, PSCs were fixed and permeabilized with 4% paraformaldehyde and 0.1% Triton X-100 at 4°C for 20 min. After washing twice with PBS, samples were blocked using 10% FCS (Sigma-Aldrich) in PBS at 4°C for 1 h. Subsequently, primary antibodies against αSMA (1:200; A2547, RRID: AB_476701; Sigma-Aldrich, Merck KGaA), vimentin (1:500; 10366-1-AP, RRID: AB_2273020; Proteintech), TRPV4 (1:200; ACC-034, RRID: AB_2040264; Alomone, RRID: AB_2534069) and Talin-1 (1:500; T3287, RRID:AB_477572; Sigma-Aldrich) were added to the gels at 4°C for 2 h. After washing three times with PBS, Alexa Fluor 488-conjugated secondary antibodies against mouse IgG (1:500; RRID: AB_2534069, Invitrogen) and Cy3-conjugated antibodies against rabbit IgG (1:500; RRID: AB_10563288, Invitrogen), as well as DAPI (1:100,000, Sigma-Aldrich), were added at 4°C for 20 min. An Axio Observer Z.1 inverted microscope (Carl Zeiss AG) equipped with a CMOS camera and 40× objective (400× total magnification, Carl Zeiss AG) objective was used to acquire the immunofluorescence images.

Piezo1 channel staining was performed adhering to the protocol by Waschk et al. (2011). Gradient hydrogels were washed with PBS then blocked with 1% BSA at 4°C for 1 h. Primary antibody against Piezo1 (1:200; 15939-1-AP, RRID: AB_2231460; Proteintech) was added at 4°C for 2 h. Subsequently, the cells were fixed using 3.5% paraformaldehyde at 4°C for 20 min. Next, secondary antibody (Cy3-conjugated antibodies against rabbit IgG, 1:500; Invitrogen) and DAPI were added at 4°C for 20 min. Piezo1 fluorescence was recorded using a DMI 6000 confocal microscope from Leica Microsystems. Images were acquired with a 63× oil immersion objective and Leica LAS-X software. To determine the Piezo1 distribution as well as the channel density, the number of ion channels was collected in 5 µm^2^ regions. Where possible, four regions in the center of the cell and eight regions at the cell edges (periphery) were evaluated per cell. Moreover, background was subtracted from the cellular signal by counting stained structures in 5 µm^2^ regions outside the cell borders. To verify whether a structure was an ion channel, a line scan was placed through the long axis of the corresponding object in ImageJ (National Institutes of Health, Bethesda, USA). A structure was scored as a channel if the associated half-maximal intensity signal extended over an apparent distance of <4 pixels (<0.72 µm).

Piezo1^WT^ and Piezo1^GFAP^ KO PSCs were fixed with 4% paraformaldehyde for 10 min at room temperature and then treated with 0.1% Triton X-100 (Swain and Liddle, 2021). Cells were immunostained with a chicken anti-GFAP antibody (1:300; ab4674, RRID:AB_304558; Abcam) or rabbit anti-Cre antibody (1:500; NB100-56133, RRID:AB_838060; Novus Biological) at 2–8°C. Secondary antibodies included DyLight 488-conjugated anti-chicken IgG (1:1000; 703-546-155, RRID: AB_2340376; Jackson ImmunoResearch) and Cy 3-conjugated anti-rabbit IgG (1:1000; 111-165-003, RRID: AB_2338000; Jackson ImmunoResearch), used for 1 h at room temperature. All images were captured with a Zeiss Axio observer Z1 with a 20× objective.

### Intracellular Ca^2+^ measurements

For intracellular Ca^2+^ measurements, the ratiometric Ca^2+^ indicator dye 3 µM Fura-2 AM (Cayman Chemical) was used. The measurements were made using an inverted Axiovert 200 M microscope from Carl Zeiss AG equipped with a pco.edge 5.5 camera (PCO AG). The images were taken at 40× objective magnification. The VisiChrome High-Speed Polychromator System (Visitron Systems GmbH) was used as the light source, generating monochromatic light at wavelengths 340 nm and 380 nm for Fura-2 excitation. The images were taken exclusively in the 2×2 mm square where the presence of the stiffness gradient had been verified using the AFM technique. A gel region was selected in which the Ca^2+^ concentration of the cells was measured over an 8 min period. For this purpose, recordings were made in 10 s intervals using both excitation wavelengths. First, the glass-bottom dishes were superfused for 2 min with Ca^2+^-free solution (122.5 mM NaCl, 0.8 mM MgCl_2_, 5.4 mM KCl, 10 mM HEPES, 1 mM EGTA), to identify stressed or dying cells, before switching back to HEPES-buffered Ringer's solution (122.5 mM NaCl, 1.2 mM CaCl_2_, 0.8 mM MgCl_2_, 5.4 mM KCl, 10 mM HEPES, 5.5 mM glucose) for 1 min. This was followed by a switch to a Ringer's solution containing 5 µM Yoda1 for 5 min. ImageJ 1.52p software was used to evaluate the measurements. First, a pixel-per-pixel background correction was performed, as well as the application of a Gaussian Blur filter with a radius of 200 pixels to reduce background noise. The absolute emission intensity values of the pixels at both excitation wavelengths were used for the subsequent pixel-by-pixel F_340_/F_380_ ratio generation. The resulting 32-bit floating-point ratio image was used for all subsequent evaluations. The increase in the F_340_/F_380_ ratio served as a measure of the increase in intracellular Ca^2+^ concentration upon Yoda1 administration.

For Piezo1^WT^ and Piezo1^GFAP^ KO PSCs, cell Ca^2+^ imaging was performed on quiescent PSCs cultured on the Matrigel-coated plates using Calcium 6-QF dye (Molecular Devices), as previously described (Swain and Liddle, 2021; Swain et al., 2022). Cells were imaged in HBSS buffer (PAN Biotech) with 2 mM Ca^2+^. Imaging was performed using a Zeiss Axio observer Z1 with a 20× objective at room temperature, and the intensity of Calcium 6-QF over time was analyzed using MetaMorph image processing and analysis software (Molecular Devices). Faintly and highly fluorescence-loaded cells were excluded from the analysis. The chemicals used in Ca^2+^ imaging experiments included Yoda1 (Tocris, cat. No. 5586).

To assess the rate of Ca^2+^ influx into PSCs, we applied the Mn^2+^ quench technique (Fels et al., 2016; Kuntze et al., 2020). Mn^2+^ enters cells via similar pathways as Ca^2+^. However, unlike Ca^2+^ ions, Mn^2+^ ions quench the fluorescence emission of the Ca^2+^-sensitive dye Fura-2. PSCs were loaded with 3 μM Fura-2 AM for 30 min with DMEM/Ham's-F12 in the incubator. The experiments were performed at the Ca^2+^ insensitive, isosbestic excitation wavelength of Fura-2 (365 nm), and fluorescence emission (F_365_) was recorded at 510 nm. Images were acquired in 5 s intervals. During measurements, a 5 min control period with Ringer's solution was followed by a 5 min superfusion of Mn^2+^-containing Ringer's (Mn^2+^ Ringer's) solution. The Mn^2+^ concentration in Mn^2+^ Ringer's was 200 µM. Data analysis was performed by measuring fluorescence intensities over the whole cell area and correcting it for background fluorescence. For data analysis, the intensity was normalized to the values of the control period. Afterwards, regression analysis of the Ca^2+^-dependent Fura-2 AM fluorescence intensity over time allowed the determination of the change of fluorescence quenching. The corresponding mean change of the slope (Δm F_365_/t=m2−m1) was calculated (with m1 being the slope during control conditions and m2 being the initial slope during Mn^2+^ perfusion). Lastly, for easier interpretation, the inverse value of the Mn^2+^ quench was determined, which is a surrogate of Ca^2+^ influx.

### Numerics

#### Finite-volume discretization

The system comprising Eqn 3a and Eqn 3b was numerically solved by a finite volume scheme that is based on the Scharfetter–Gummel flux approximation and is described in more detail below. The implementation is based on Julia (Bezanson et al., 2017). The simulations in Fig. 5 use 16,384 time steps with a step size of 1/5120 on a grid with a spacing of 1/512.

The numerical simulation of the system comprising Eqn 3a and Eqn 3b, which can also be expressed as
(4rma)

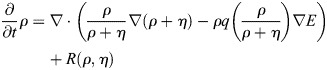

(4rmb)

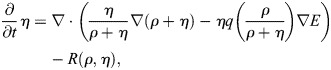
is based on a finite volume scheme described below. Here *ρ*=*ρ*(*x*, *t*) and *η*=*η*(*x*, *t*) denote the densities of the open and closed channels on the cell, respectively. *E* denotes the elastic modulus of the substratum (i.e. it is larger for a stiffer material). The function *q* accounts for the cell's strength of sensing the gradient of *E* due to the relative number of open versus closed channels. *R*(*ρ*, *η*) describes the combined opening and closing of the channels. In our mathematical model we defined *R*(*ρ*, *η*)=*αE* · *η*−*βρ*.

The proposed numerical scheme to solve the above system is based on the Scharfetter–Gummel flux approximation originating from Scharfetter and Gummel (1969), where the authors construct a numerical scheme for a system modeling semiconductor devices. Their objective was to develop a robust scheme for discontinuities or rapid variations in the potential. Independently, the same type of flux was introduced in Il'in (1969) for finite-difference schemes. The Scharfetter–Gummel scheme became one of the preferred finite-volume schemes for drift-diffusion equations. While the original scheme deals with a spatially one-dimensional problem, it has been generalized to higher dimensions (Farrell and Gartland, 1991; Gajewski et al., 2003), and the flux discretization is the basis for numerous other generalizations, such as those for equations with non-linear diffusion (Jüngel, 1995; Bessemoulin-Chatard, 2012; Eymard et al., 2006) and to systems with source terms (Cheng and Boonkkamp, 2021; Ten Thije Boonkkamp and Anthonissen, 2011). The Scharfetter–Gummel flux approximation has previously been applied in the context of chemotaxis and aggregation equations (Zhou and Saito, 2017; Liu et al., 2016; Almeida et al., 2019; Shen and Xu, 2020; Schlichting and Seis, 2020; see also the review Bärwolff and Walentiny, 2018).

First, we generalize our problem to:
(5rma)

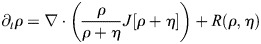

(5rmb)

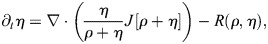
which is in flux form. Then we set 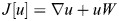 for some given (possibly time-dependent) vectorfield *W*. This will later be defined as a numerical approximation of 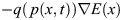. Hence, the above driving flux *J* depends only on the sum *u*=*ρ*+*η* and space and time.

The notation for finite volume schemes is as follows (see also Eymard et al., 2000). Consider a polyhedral tessellation 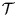 of the domain Ω with volumes 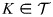 and centers *x*_*K*_.

Neighboring cells are denoted by *L*∼*K* with common face 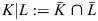.

Note: here cells are a technical expression for so-called numerical cells in finite volume schemes, which have nothing to do with the biological cells we are considering in this paper.

The distance between cell centers is denoted with *d*_*KL*_:=|*x*_*k*_−*x*_*L*_|.

The transmission coefficient is 
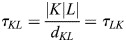
.

We use |*K*| to denote the *d*-dimensional volume and, by slight abuse of notation, we denote by |*K*|*L*| the *d*−1-dimensional surface area.

We use *ρ*_*K*_, *η*_*K*_ for the cell averages of densities and set *u*_*K*_=*ρ*_*K*_+*η*_*K*_.


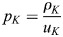
 and 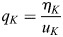 denote the relative weights of the two densities.

For *τ*∈[0, 1], *ρ*^*n*+*τ*^ denotes the linear interpolation between *ρ*^*n*^ and *ρ*^*n*+1^.

This allows us to define the full range of explicit to implicit schemes at the same time. Given some flux approximation *J*_*KL*_[*u*^*n*+*τ*^], which will be specified later, we introduce the scheme:
(6rma)

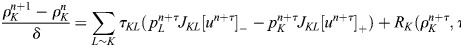

(6rmb)

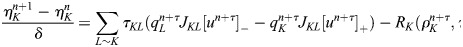
where (*a*)_+_=max{*a*, 0} and (*a*)_−_=(−*a*)_+_ denote the positive and negative part, respectively. Our reaction term is explicitly given by:

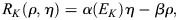
with *E*_*K*_ being the cell average of the elastic modulus *E*.

It is left to find approximations for the normal fluxes 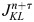 across boundary faces. If we assume that some discretization 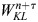 of *W* (depending in our case on 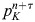) is given, then the normal component of the flux *J*[*u*] in Eqn 5a and Eqn 5b can be approximated by the cell problem (for example, see Scharfetter and Gummel, 1969; Jüngel, 1995; Farrell et al., 2017; Schlichting and Seis, 2020):

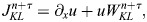
where *u* is the unknown along the line segment [*x*_*k*_, *x*_*L*_]:={(1−*s*)*x*_*k*_+*sx*_*L*_:*s*∈[0, 1]} with boundary conditions 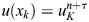 and 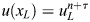.

This is the classical Scharfetter–Gummel interpolation, and by setting *d*_*KL*_=|*x*_*K*_−*x*_*L*_| we obtain the flux 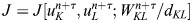 given by:
(7)

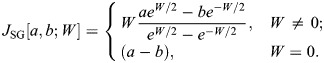


Hence, we can close the scheme by setting:
(8)

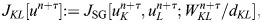
with 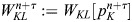 given by:
(9)

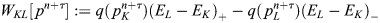


#### Simulation

For the simulation in Fig. 5, we implemented an explicit version (*τ*=0) of the scheme comprising Eqn 6a and Eqn 6b on the interval [0, 1] with a uniform tessellation. That is, for given *h*=1/*N*, we set 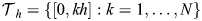 and *x*_*K*_=(*k*+1/2)*h* for *k*=0, …, *N*−1.

We have the two discrete continuity equations:

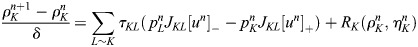


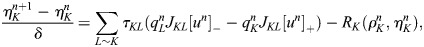
where we impose a no-flux boundary condition, that is *J*_0,1_=0=*J*_*N*,*N*+1_, and the sum over *LK* consists of the two addends *K*−1 and *k*+1.

Hereby, in one dimension *τ*_*KL*_=*h*^−1^_,_ 
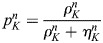
 and 
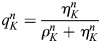
.

For the implementation, we choose *R*_*K*_(*ρ*, *η*)=*αE*_*K*_*η*−*βρ* with *E*_*K*_=*E*(*x*_*K*_).

For the simulation we use an elasticity profile on [0, 1] resembling the elasticity modulus of the experimental made substrate given by:

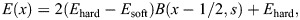
where *B*(*y*, *s*)=*y*/(exp(−*sy*)−1). In the simulations in Fig. 5, *E*_hard_=0.1, *E*_soft_=0.01 and the scale parameter *s*=30. We use the Scharfetter–Gummel flux interpolation (Eqn 8) in explicit form:

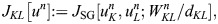
with *J*_SG_ given in Eqn 7 and upwind discretization of the chemical potential given in Eqn 9 by:

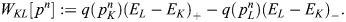


Hereby, the discrete mobility is 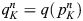, with the continuous mobility function given in terms of a bell-shaped function with compact support by:

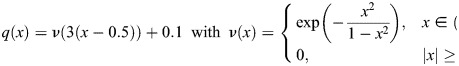


### Statistical analysis

All data sets were tested for normal distribution using Anderson–Darling and D'Agostino–Pearson tests. For normally distributed data sets, mean and s.e.m. were used to represent the measured values. An unpaired *t*-test was used as a significance test for two comparison groups. In the absence of a normal distribution, the median was mapped, and the 95% confidence interval was mapped as the measure of dispersion. The Mann–Whitney test was used as a hypothesis test for two comparison groups, and the Kruskal–Wallis test for multiple comparisons.

All statistical analyses were performed as two-sided tests at a significance level α of 0.05. Dunn's test was used as a post hoc test in the absence of a normal distribution.

## Supplementary Material

10.1242/joces.263846_sup1 Supplementary information
