# Supplementary material for: Piezo1-induced durotaxis of pancreatic stellate cells depends on TRPC1 and TRPV4 channels
Source: J Cell Sci. 2025 Apr 25;138(8):jcs263846. doi: 10.1242/jcs.263846 (PMC12136172; doi:10.1242/jcs.263846)
Supplement: Supplementary information [file joces-138-263846-s1.pdf]

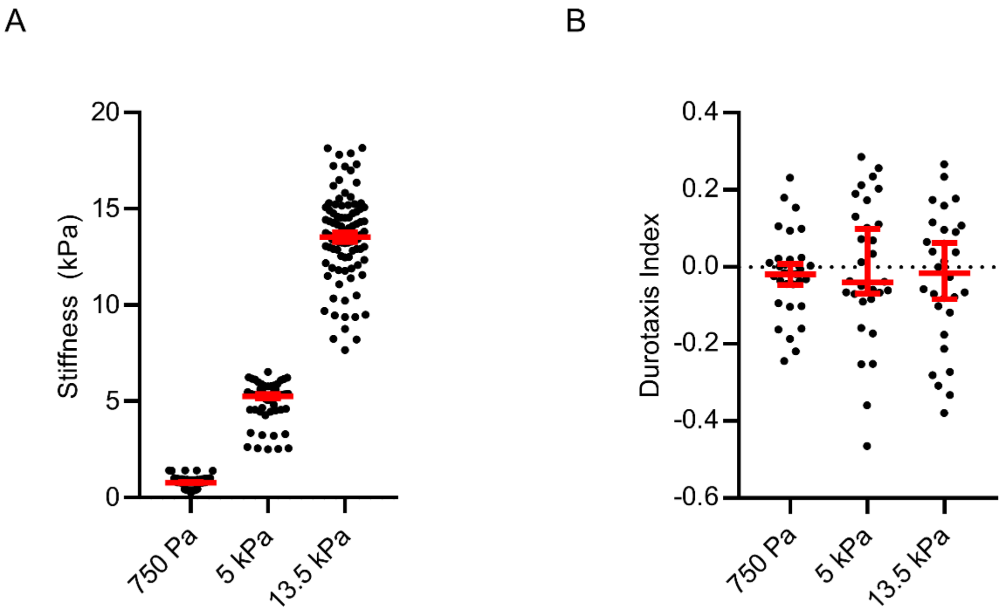

Fig. S1. PSCs on hydrogels with constant stiffness migrate non-directionally

A) Scatter plot shows homogeneous gel stiffnesses measured from n≥10 points of N=5 gels. **B)** Scatter plot indicates durotaxis indices of PSCs derived from the trajectories in Fig. 1B. (n=30 PSCs in N=3 independent experiments). Data in (A) is mean ± SEM and in (B) median ± 95% CI. Statistical test in (B) is one-sample t-test.

Journal of Cell Science • Supplementary information

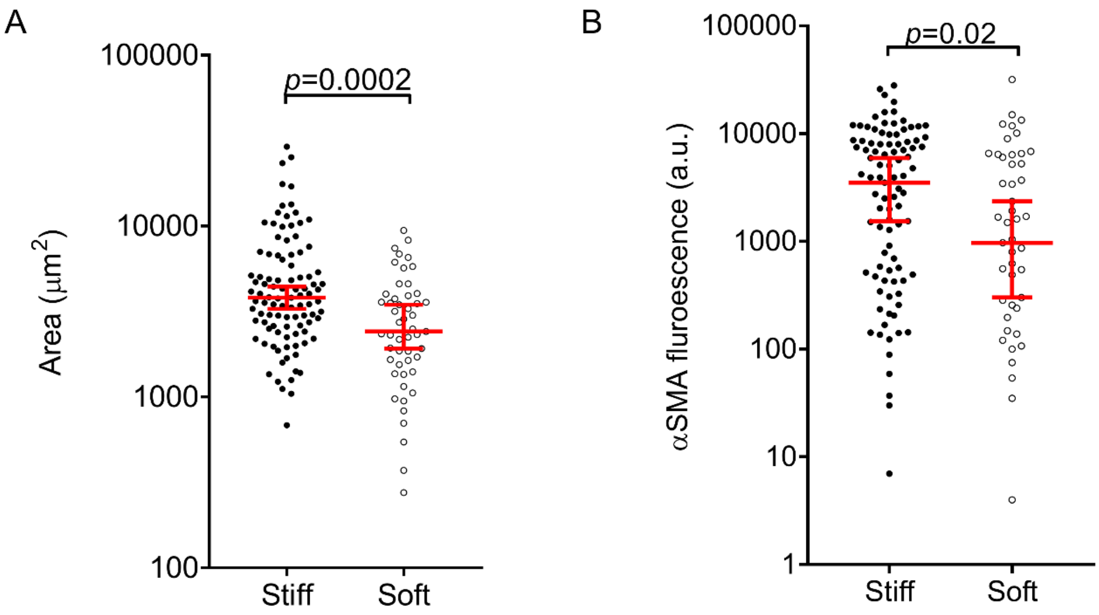

Fig. S2. PSC immunostaining reveals higher cell area and αSMA intensity on a stiffer substrate

A) Scatter plot shows cellular areas derived from αSMA-stained PSCs depicted in Fig. 2E. **B)** Scatter plot of αSMA fluorescence intensity of PSCs on the stiff and soft parts of the gradient hydrogel. n cells measured / N experiments ≥ 51/4. Data in (A) and (B) are median ± 95% CI. Statistical test are Mann-Whitney U-tests.

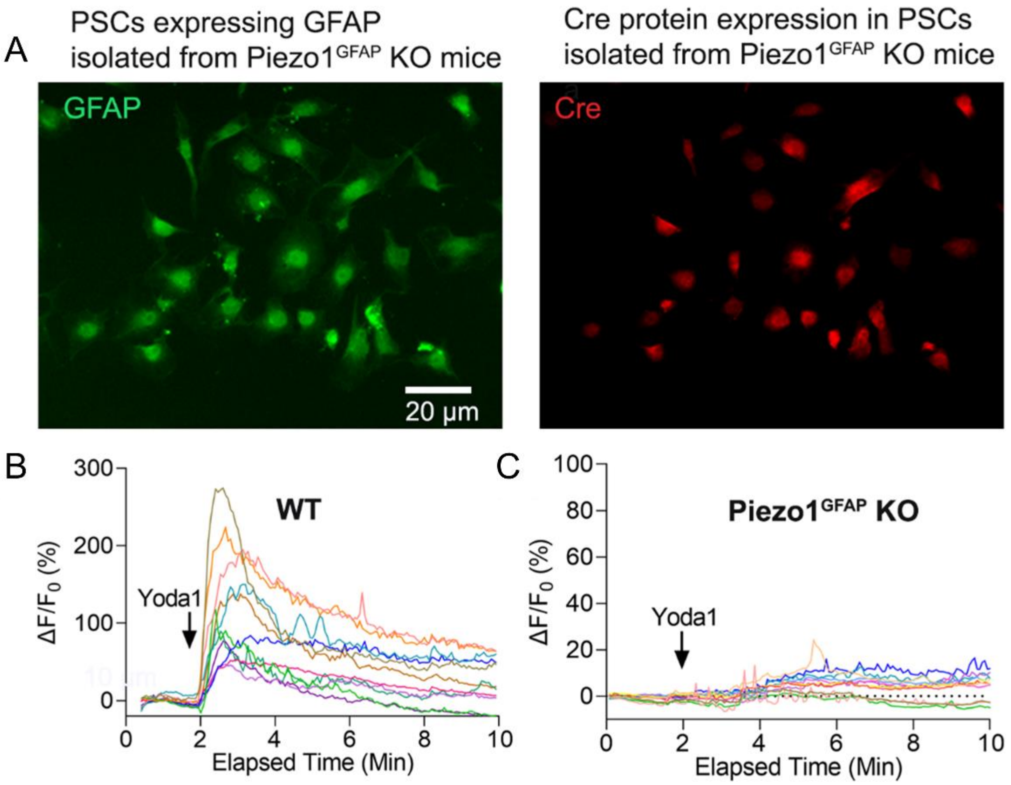

Fig. S3. Piezo1 deletion in GFAP expressing mouse PSCs

A) Images showing PSCs isolated from mouse line B6. Cg-Tg (GFAP- cre/ERT2; *Piezo1$^{fl/f}$)* after tamoxifen injection (referred to as Piezo1^{GFAP} KO mice) cultured for 3 days in a Matrigel-coated plate as described previously (1), expressed both stellate cell marker GFAP (green) and Cre protein (red). Scale bar: 20 µm. **B)** and **C)** Piezo1 agonist Yoda1 (5 µM) induces an elevation of the intracellular Ca^{2+} concentration in PSCs isolated from wild type but not from Piezo1^{GFAP} KO mice, confirming that successful deletion of Piezo1 in GFAP expressing PSCs, n=12 cells. Each trace represents Yoda1-induced single cell relative fluorescence intensity ($\Delta F/F_0$) of the calcium 6-QF dye over time. ΔF is the change in fluorescence intensity (F-F_0), and F_0 is the basal fluorescence intensity before Yoda1 application.

Journal of Cell Science • Supplementary information

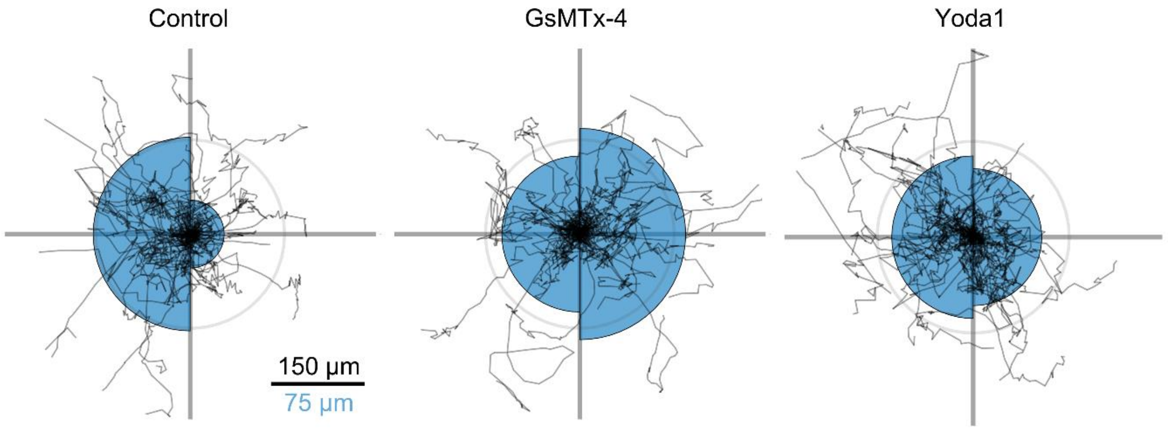

Fig. S4. Durotaxis polar plots of PSCs with pharmacological Piezo1 modulation

The durotaxis plot data aims to support Fig. 3C. Durotaxis polar plots depict individual PSC trajectories over 24 h (black lines). The radius of the blue half circles is proportional to the mean cellular displacement into the directions 0° and 180°, respectively. Radial lines indicate 0°, 90°, 180°, 270°.

Journal of Cell Science • Supplementary information

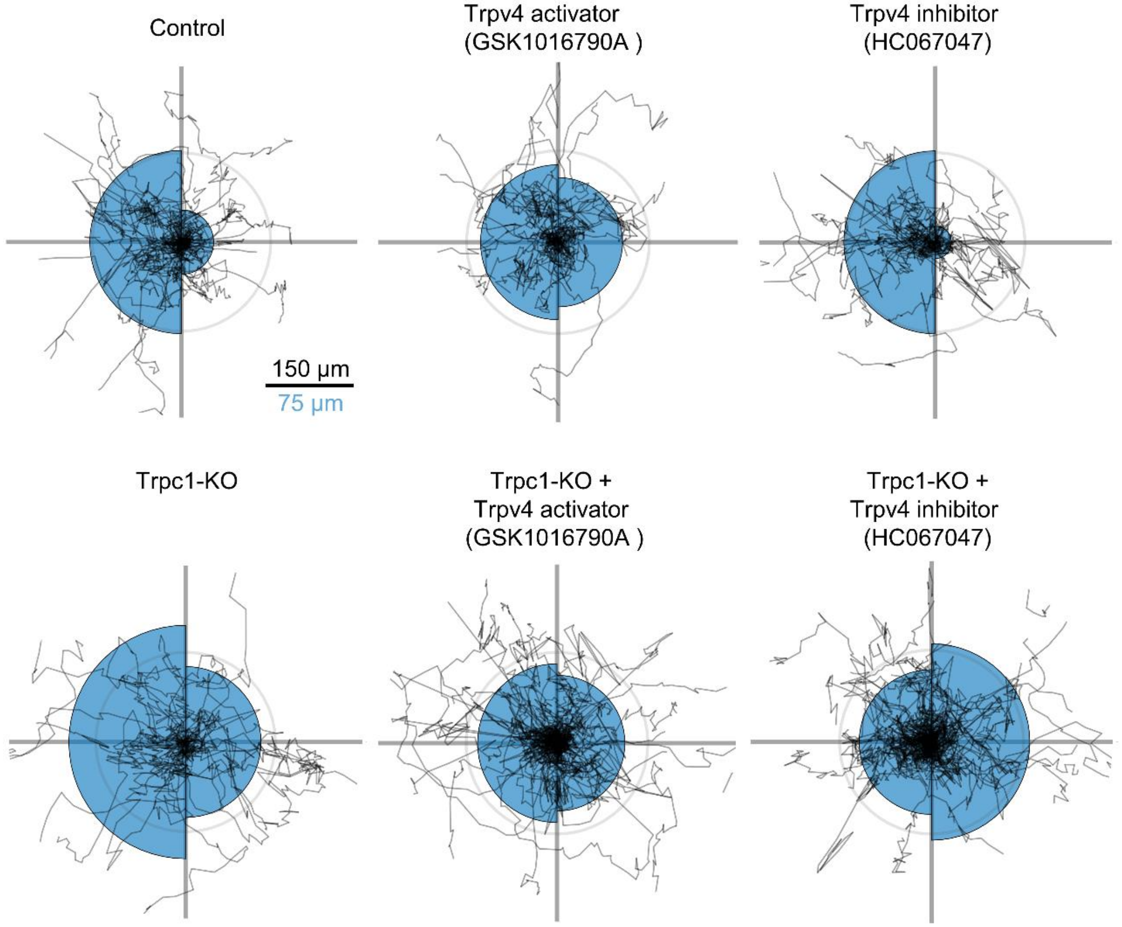

Fig. S5. Durotaxis polar plots of PSCs with TRPV4 and TRPC1 modulation

The durotaxis plot data aims to support Fig. 5A. Durotaxis polar plots depict individual PSC trajectories over 24 h (black lines). The radius of the blue half circles is proportional to the mean cellular displacement into the directions 0° and 180°, respectively. Radial lines indicate 0°, 90°, 180°, 270°.

Journal of Cell Science • Supplementary information

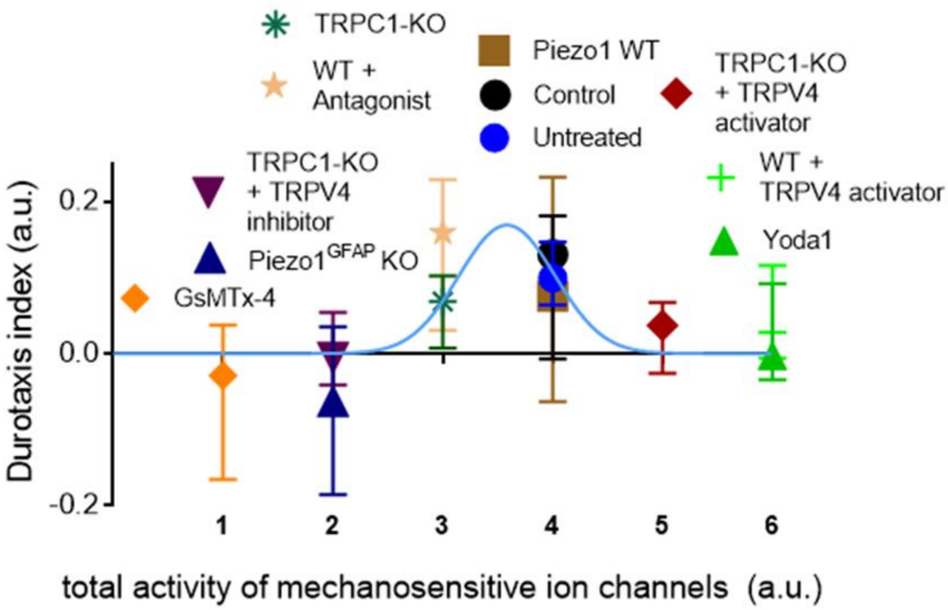

Fig. S6. Durotaxis requires intermediate activity of mechanosensitive ion channels

The depicted data shows details regarding Fig. 5C. Scatter plot shows the durotaxis index as a function of the total ion channel activity, derived from Fig. 5A and B. Inhibitor and activator refer to TRPV4 modulation with HC-0606006 and GSK101489, respectively. The blue line corresponds to a gaussian curve fit from Fig. 5c, indicating the bell-shaped relationship between channel activity and durotaxis. Data points are median ± 95% CI.

Journal of Cell Science • Supplementary information

Table S1. Acrylamide mixture used for hydrogels with constant stiffness

Acrylamide (%)	Bisacrylamide (%)	Hydroxyacryl-amide (%)	Volume Acrylamide mix (µl)	Volume PBS (µl)	Stiffness
2,5	0,07	0,8	53	447	750 Pa
3,54	0,1	1,15	75	425	5 kPa
7,08	0,2	2,3	150	350	13.5 kPa

Table S2. Statistical comparisons between individual groups for Figs. 3B and 4B.

**Fig. 3C**					
Dunnett's multiple comparisons test	Mean Diff,	Summary	Adjusted P Value	n1	n2
**Constant stiffness vs. Piezo1 Wt**	**-0.1257**	*****	**0.0118**	**89**	**30**
Constant stiffness vs. Piezo1 cKO	0.05363	ns	0.6706	89	22
**Constant stiffness vs. Gradient stiffness**	**-0.1036**	*****	**0.0136**	**89**	**50**
Constant stiffness vs. GsMTx (100nM)	0.02666	ns	0.9094	89	48
Constant stiffness vs. Yoda1 (5µm)	-0.02607	ns	0.9145	89	50

Journal of Cell Science • Supplementary information

**Fig. 4B**					
Dunn's multiple comparisons test	Mean rank diff.	Summary	Adjusted P Value	n1	n2
**Constant stiffness vs. Gradient stiffness**	**-62.59**	**	**0.0051**	**89**	**50**
Constant stiffness vs. Trpc1 WT + Trpv4 activator	-47.76	*	0.0369	89	30
**Constant stiffness vs. Trpc1 WT + Trpv4 inhibitor**	**-90.76**	***	**0.0001**	**89**	**28**
**Constant stiffness vs. Trpc1 KO**	**-42.19**	*	**0.0484**	**89**	**59**
Constant stiffness vs. Trpc1 KO + Trpv4 activator	-25.28	ns	0.1627	89	60
Constant stiffness vs. Trpc1 KO + Trpv4 inhibitor	-10.6	ns	0.5603	89	59
Gradient stiffness vs. Trpc1 WT + Trpv4 activator	14.83	ns	0.5535	50	30
Gradient stiffness vs. Trpc1 WT + Trpv4 inhibitor	-28.16	ns	0.271	50	28
Gradient stiffness vs. Trpc1 KO	20.4	ns	0.3275	50	59
Gradient stiffness vs. Trpc1 KO + Trpv4 activator	37.32	ns	0.0722	50	60
**Gradient stiffness vs. Trpc1 KO + Trpv4 inhibitor**	**51.99**	*	**0.0166**	**50**	**59**
Trpc1 WT + Trpv4 activator vs. Trpc1 WT + Trpv4 inhibitor	-43	ns	0.1312	30	28
Trpc1 WT + Trpv4 activator vs. Trpc1 KO	5.568	ns	0.8188	30	59
Trpc1 WT + Trpv4 activator vs. Trpc1 KO + Trpv4 activator	22.48	ns	0.3536	30	60
Trpc1 WT + Trpv4 activator vs. Trpc1 KO + Trpv4 inhibitor	37.16	ns	0.1263	30	59
Trpc1 WT + Trpv4 inhibitor vs. Trpc1 KO	48.57	ns	0.0509	28	59
Trpc1 WT + Trpv4 inhibitor vs. Trpc1 KO + Trpv4 activator	61.48	**	0.0083	28	60
Trpc1 WT + Trpv4 inhibitor vs. Trpc1 KO + Trpv4 inhibitor	80.16	**	0.0013	28	59
Trpc1 KO vs. Trpc1 KO + Trpv4 activator	16.91	ns	0.3947	59	60
Trpc1 KO vs. Trpc1 KO + Trpv4 inhibitor	31.59	ns	0.1134	59	59
Trpc1 KO + Trpv4 activator vs. Trpc1 KO + Trpv4 inhibitor	14.68	ns	0.4602	60	59

Table S3. Summary of one-sample Wilcoxon tests of durotaxis experiments, compared to zero directionality (theoretical median = 0).

Wilcoxon Signed Rank Test	Theoretical median	Actual median	Sum of signed ranks (W)	P value
Constant stiffness	0	-0.02688	-529	0.2816
**Piezo1 Wt**	**0**	**0.07547**	**143**	**0.0367**
**Piezo1^{GFAP} KO**	**0**	**-0.06296**	**-133**	**0.0301**
**Gradient stiffness**	**0**	**0.1303**	**521**	**0.0112**
GsMTx (100nM)	0	-0.02914	-292	0.1366
Yoda1 (5µm)	0	-0.002708	99	0.639
Trpc1 WT + Trpv4 activator	0	0.02781	169	0.0841
**Trpc1 WT + Trpv4 inhibitor**	**0**	**0.1587**	**278**	**0.001**
**Trpc1 KO**	**0**	**0.06848**	**534**	**0.0437**
Trpc1 KO + Trpv4 activator	0	0.03681	312	0.2543
Trpc1 KO + Trpv4 inhibitor	0	-0.004847	-20	0.9433

Journal of Cell Science • Supplementary information
